# How can environmental degradation and income disparities influence national health: an eye bird view on China’s provinces

**DOI:** 10.3389/fpubh.2023.1094775

**Published:** 2023-07-07

**Authors:** Zhanqiang Shao, Lingling Dou

**Affiliations:** ^1^School of Finance, Nankai University, Tianjin, China; ^2^School of Statistics and Big Data, Henan University of Economics and Law, Henan, China

**Keywords:** mortality rate, carbon emission, renewable energy, ICT, income inequality

## Abstract

Growing socio-economic disparity is a global issue that could disturb community health. Numerous case studies have examined the health influences of income disparities as well as the patterns that implicate those disparities. Therefore, this study attempts to examine the core determinants of mortality rate, which are environmental degradation, green energy, health expenditures, and technology (ICT) for the 25 provinces of China over the period of 2005–2020. This study uses a series of estimators to investigate the preferred objectives in which CS-ARDL and common correlated effect mean group (CCE-MG). Estimated results show the significant contribution of environmental deterioration and income inequality to the mortality rate. Furthermore, health expenditures, ICT, and green energy significantly reduce the mortality rate. Similarly, the moderate effect of income inequality on health expenditure, green energy, and ICT significantly reduces the mortality rate in selected provinces of China. More interestingly, the current study suggests policy implications to reduce the rising trend of mortality rate.

## 1. Introduction

Air pollution has become one of the world’s main public health issues, with millions dying from linked ailments each year (1). In China alone, outdoor air pollution is accountable for more than 1 million early casualties in 2016, according to the World Health Organization. Growing the heart disease, risk of stroke, lung cancer, and long-lasting and severe respiratory disorders, PM10 and PM2.5 are particularly harmful, as noted by Chen et al. (2). The burning of fossil fuels, which has characterized economic growth in China over the past four decades (3), contributes to the country’s already severe air pollution problem (4). Air pollution is a major social problem that has far-reaching consequences (5). This rising trend has been observed in response to global growth activities.

China’s economic growth since the restructuring and opening up is well-known, and the country is currently the world’s second-biggest economy (6). However, a number of environmental pollution concerns have emerged during this rapid development, endangering not only the natural ecosystem and ecological balance but also people’s health (7). Approximately 10% of China’s GDP could be lost to environmental pollution and ecological destruction, according to a report titled “Environmental Protection in China (1996–2005).” China scored 50.74 on Yale University’s and Columbia University’s Environmental Performance Index Report (2018), enlisting it 120th overall and 177th in air quality. According to the bulletin’s second report on China’s ecological situation, 70.7% of China’s metropolises (or 239 of the 338 prefecture-level cities) have polluted air (4). The Chinese government and population have come to recognize the critical need to protect the environment in the face of worsening environmental pollution and ecological destruction (8). The Chinese government has used a wide variety of administrative policies, economic measures, and market processes to curb pollution to free up scarce resources and improve the environment (9). Although some progress has been made thanks to these initiatives, the severity of China’s environmental pollution problem persists (10, 11). For this reason, China’s objective of green and sustainable growth will be impossible unless severe issues like resource scarcity, pollution, and ecological degradation can be substantially addressed (9). Similarly, Chien et al. (12) used a comparative study of China and the USA and found the environmental impact on health positive for China while negative for the USA. However, the green patents decrease the level of emissions, but they ignore discussing the level of growth and emissions in China (13).

Besides the economic growth activities and their impact on environmental pollution, income disparity is also a leading cause of health issues for human beings. Income inequality is the term used to describe the unequal distribution of income among individuals across regions, states, or nations (14). It is generally acknowledged that ecologically speaking, wealth inequality has an impact on health outcomes. Our attention is on the more debatable claim that there is a link between economic disparity and health, even after considering individual income (15). Those who work in public health and policy are quite interested in this connection (16), yet findings from studies examining the connection have been contradictory (17). Recent research assessing the correlation between income inequality and health found that the results of 13 different systematic studies were all over the map (18). There is still no conclusive evidence linking income disparity to health outcomes, as seen by the large statistical heterogeneity in these reviews. Causal evaluation relies heavily on understanding the factors contributing to variation in effect sizes between studies (i.e., statistical heterogeneity or transportability in causal inference) (19).

A very ambiguous debate has been observed on the mortality rate. Similarly, the existing studies have not intensely focused and have not tried to introduce the leading indicators of mortality rate. Therefore, the current study introduces some interesting variables and tries to fill this gap. This study has the following contributions to the existing literature. It is hypothesized that the impact of state-level income inequality is more likely to manifest after several years rather than instantly, so the health sector is being taken into account for this study to investigate the varying responses of variables and their dependence on prolonged exposure to unequal distribution of income within a population. Some researchers have examined the correlation between income inequality and mortality rates in specific Chinese provinces between 2005 and 2020. Given the crucial importance of the economic conditions within the society as young women may or may not enter family planning stages in selected regions; therefore, it would guide the policymakers to reshape their health policies to control the severe challenges. Similarly, rising CO_2_ emissions negatively impact various health indices, reducing human capital productivity and slowing the rate of social and economic growth. The child mortality rate is one of the most important health indicators. Pollution levels are rising to alarming levels all around the world. Infant mortality, cardiovascular disease, pulmonary disease, allergies, increased oxidative stress, endothelial dysfunction, mental illness, and other negative health impacts can begin at a young age due to environmental pollutants. Therefore, the current study tries to discuss environmental degradation and its harmful impact on the mortality rate.

Furthermore, this study proposed a significant factor that may inform pregnant women how to secure their children. Therefore, we develop a well-organized model to understand one of the critical drivers of digitalization: information and communication technology (ICT) use and its impact on the mortality rate. Thus, it may guide policy analysts to interlink their health policies with digitalization to secure a mother from this severe challenge. Very interestingly, health expenditures by the government can save from this trap to a mother. In light of this, we examine how public health spending affects health outcomes in the context of China’s provinces, concentrating on one particular health outcome, namely the death rate. There are two reasons for focusing: (1) As we go into greater detail. However, state and federal governments share responsibility for health. The former is much more involved in allocating funds for the delivery of health services. In these provinces, state governments primarily provide health services, including medical care and public health initiatives. However, China has made a significant contribution to the health sector. In order to quantify its impact on health outcomes, this may lead to differences in public health spending among provinces. (2) The data on public health expenditure and other important factors is largely consistent and comparable, which is an advantage of researching states inside China provinces. As a result, provinces-level analysis enables us to avoid numerous challenging problems with data comparability, which become crucial in cross-province contexts. Furthermore, using green energy in daily human and economic activities causes less harm to the environment and causes a decline in health issues. In addition, the primary goal of this study is to inspect the moderating effect of wealth disparity on mortality rate by analyzing the correlation between health spending, green energy, and ICT.

In order to compile above contribution to existing literature, the current study tries to demonstrate the lnkage of environmental pollution toward the mortality rate because China is in the top three polluted economies. Therefore, it is necessary to investigate the significant impact of increasing CO_2_ emissions on mortality rate and try to answer whether it harm to pregnant women and their child’s health. Similarly, income inequality has become a serious challenge for the globe and especially in developing and emerging economies. Over the time it has seen the income disparity increases and richer become more richer. Due to income inequality, to access the health facilities may vary in different income classes. In simple words, China’s government has a keen attention to their health sector, beside, the government initiative it is necessary to investigate whether income disparities may decline or increase the mortality rate. Since the last three decades, China’s government has made best initiatives toward education, health and other social welfare aspects; therefore, this study also tries to investigate the key role of health expenditures in diminish the mortality rate. Basically, this study makes a significant testation of Chinese suggested policies and would answer whether implemented policies significantly reduces mortality rate or not. Finally, the penetration of ICT to human and economic activities has a significant importance and ICT has tried to create an ease; therefore, this study also investigate the main and moderate role of ICT on mortality rate and validates the policy implications suggested by China’s government. This work uses a novel set of econometric approaches to achieve its aims, including tests for unit root dependency and co-integration as well as the CS-ARDL and CCEMG estimators for both the short and long term.

## 2. Literature review

Similarly, the literature section is a leading part of every study, which provides a brief overview of what has been done in the past. Therefore, the current study divides this section into two sub-sections such as (1) Studies on Income Inequality and Health Issues; (2) Studies on Environmental degradation and Health Issues. However, these two sub-sections details information is given as follows.

### 2.1. Studies on income inequalities and health issues

Women’s health may be more affected by socio-economic inequality and country-level policies than men’s health because they affect access to services and resources that are particularly important to women’s lives (such as prenatal care, adolescents’ health care, affordable housing, and family leave) (20). Rehman et al. (21) and others revealed strong correlations between area-level socio-economic circumstances and women’s BMI, self-rated health, elevated levels of risk behaviors connected to their health, and death (22). According to a recent systematic review, women experience a stronger negative influence of wealth inequality on their mental health than males, putting them at greater risk of mental diseases (23). Overall, because they are more possible than males to be socioeconomically poor, have offspring, and use the healthcare system, women are more susceptible to the negative outcomes of income inequality and the inadequate division of public resources (24).

Only a small number of researchers have looked at how maternal, and fetal outcomes are impacted by wealth disparity. According to a cross-sectional study in the US, women with small children are more likely to experience poor physical and mental health when there is considerable state-level wealth inequality (25). Preterm birth and low birth weight were more likely in people who lived in high or medium-inequality areas at the time of conception (26). In addition, despite governing for concurrent changes in poverty, total income, and unemployment rates, a current US study found that residing in a state where income inequality widened for the year preceding delivery increased the risk of preterm birth (27). However, the effects of context on maternal mortality are poorly understood. Maternal mortality is associated with women’s education, health insurance coverage, health expenditure per person, and global poverty, according to many ecological studies (28). However, the majority of analyses that have examined related socio-economic aspects in low-and middle-income nations either failed to find a correlation between area-level maternal mortality and income inequality [see, for example, (29–31)] or overlooked unequal income distribution as an important explanatory factor (32). Delay associations have not been studied, despite the possibility that prolonged exposure to socio-economic context may have a more significant impact on maternal and perinatal outcomes (33).

Albrizio et al. (34) made a seminal addition to this field when they discovered that income redistribution improves the health of the poor more than it affects the health of the rich and hence enhances the health of the population as a whole. According to his article, “the distribution of income is a potential source of variance in the basic link between national life expectancy and average national income.” One’s health is negatively impacted by income disparity, Mohsin et al. (35), regardless of where one falls on the income scale. He proposed a psychological explanation for this phenomenon, arguing that more equitable societies foster healthier lifestyles like less smoking, less comfort food consumption, and lower stress levels overall. Inequality exacerbates tensions in social relationships by heightening class distinctions and promoting unhealthy levels of rivalry. Some negative health outcomes and accelerated aging are associated with stress (36). In health and epidemiological research, the idea that wealth inequality has a direct effect on health is known as the income inequality hypothesis.

According to these skeptics, previous findings may have been statistical artifacts; instead, the aggregate association may result from a concave relation between income and health rather than a linear one (37). Other objections focus on the reliability of the initial results. While the income inequality-health hypothesis has been studied extensively inside and across rich nations, some experts have decided that the evidence does not support it (38). Some researchers looked at what factors lead to health disparities and inequality. Caruso et al. (39), for instance, revealed that economic differences in municipalities had a negative influence on mortality in the adult population, net of individual income, by analyzing census data and other national population registers from Norway. When other unseen attributes of those cities were included, results were considerably more contradictory.

There is strong evidence for the income inequality hypothesis, notwithstanding some skepticism about the link between economic disparity and health. Titl and De Witte (40) conducted a literature assessment using a causal epidemiological approach and found that the majority of the peer-reviewed, published papers backed the claim that income disparity negatively impacts health. About 60 million respondents from 9 cohort studies and about 1.3 million people from 19 cross-sectional studies were included in a meta-analysis conducted by Kartal et al. (41). They found a “moderate” impact, which suggested that bringing the Gini coefficient in 30 OECD countries down to below 0.3 might prevent up to 1.5 million lives. El Chaarani et al. (42) found that size matters by evaluating multilevel studies examining the relationship between income and health at individual and population levels. Inequality and health are often studied at the state level, where political mechanisms and contextual consequences are simpler to isolate. Coccia (43) has also addressed this issue. They suggested that the lack of evidence for income inequality could be attributed, in part, to the use of insufficiently large analytical units to adequately account for disparities in social class and other important forms of social variability. In turn, the degree of residential segregation was found to have a greater impression on income inequality in smaller geographical units, whereas income variations within smaller neighborhoods mattered significantly less. It may not be wealth inequality within poor neighborhoods that is to blame for the poor health of its residents, but rather their marginalization from the rest of society.

### 2.2. Studies on environmental degradation and health issues

There are two main areas of study covered by the existing literature. First, the correlation between climate change and public health has increased the cost of public health services and healthcare reform. Damage to the economy and the GDP as a whole can result from citizens’ poor health (44). Green climate and carbon emissions make up the second area of study. Since studying carbon emissions and developing green technology requires a considerable investment of time and money as well as significant technological progress, this field of study has recently accelerated. Technologies that reduce carbon output are receiving much attention as the world works to create a healthier, more sustainable environment (7). Research from the past few years shows that environmental degradation has a negative influence on human health in 17 MENA countries. It can be improved by having strong environmental laws in place (45). Environmental hazards contribute to disease and health inequalities. When looking at the causes of environmental degradation, Wu et al. (46) took a look at the studies that had been conducted in the past. Depletion of natural resources is one of the most severe outcomes of environmental degradation (quality and quantity). They also looked into how this interdependence impacts people’s health all over the world. According to the research of Bashir et al. (47), shifting weather patterns significantly affect public health. It also has an impact on food production, especially in countries with a weak economy that rely heavily on agriculture (such as many of the world’s poorest nations). Consistent with previous work on the Chinese economy, these authors looked at how shifting economic conditions affect public health and environmental pollution. They used panel data to examine the connection in 30 different Chinese provinces. They validated the negative effects of pollution on public health, which has a knock-on effect on GDP per capita and acts as a barrier to fostering economic growth (48). It was found by Khan et al. (49) that environmental degradation has a positive and significant impact on healthcare costs. The bivariate association was explained using the Autoregressive distributive lag and co-integration model, which was developed using the Environmental Kuznets (EKC) hypothesis. They also noted that the direction of the relationship was uncertain and could go either up or down, depending on the level of wealth in the country. A higher real income has both short-and long-term negative effects on environmental quality (50). In the case of MENA states, it is also clear that environmental quality has a significant impact on healthcare costs. Increases in emissions lead to a corresponding increase in healthcare costs because people’s health deteriorates (51). Environmental factors, such as carbon emissions, affect the general public’s health, as Chiu and Lee (52) discovered by using the Johansen Cointegration and Vector error correction model (VECM). They added that the government should implement efficient carbon emission programs to lessen the impact on people’s health in Nigeria.

As more and more greenhouse gasses are emitted into Earth’s atmosphere, the climate is shifting. Many experts in the field of health care have noticed that fluctuating temperatures have negative effects on people’s health. Waterborne and vector-borne infections are examples of climate change-caused infectious disease transmission at the source. Sensitivity to climate plays a role in the development of Malaria, among other diseases. As a result of globalization and a lack of adequate public health infrastructure, a temperature-sensitive sickness has emerged (53). In their 1995 paper, Idrees and Majeed (54) forecasted a warmer world in which Malaria will spread to countries all over the world. Forecasts indicate that by the year 2,100, global temperatures will rise by between 1.0 and 3.5°C (55). More importantly, rising Greenhouse gas emissions are anticipated to alter Malaria transmission by changing the environment (56). Malaria in Africa was analyzed by Luo et al. (57) between 1960 and 2000. They demonstrated that Malaria spread considerably as a result of climatic changes brought on by GHG emissions. In the United States, the prevalence of diseases like Malaria and dengue fever that insects spread has been on the rise, and researchers (58) looked into what factors might be contributing to this trend. Since harsh weather is so ubiquitous, healthcare-related emissions have a negative correlation with people’s health. They proposed that enforcing health and climate rules effectively in institutions could help lessen the impact. When it comes to public health, Yang et al. (59) found that good governance is significantly important, while high-quality institutions help reduce costs. It has been found through an examination of 22 economies in sub-Saharan Africa using the method of moments (GMM) that greater governance and higher-quality institutions lead to improved health outcomes from public health spending. Although developing nations contribute relatively little to greenhouse gas emissions, Igawa and Managi (60) found that health issues have increased. From 1996 to 2016, Baloch et al. (61) analyzed the impact of African institutions. Health outcomes were found to be considerably diminished by the presence of organizational dysfunction. Environmental pollutants and institutions also significantly impact life expectancy, despite their large, positive impact on healthcare costs. They concluded that better public health and the delivery of essential services would result from increased governance and institutional excellence (62).

### 2.3. Studies on information and communication technology and health issues

With the help of ICT, we can share and disseminate data rapidly, cheaply, and across great distances (63). Evidence of ICT’s positive effects on health outcomes is abundant in the research available. In Uganda, for example, a program that used radio technology to reach pregnant women with health information had a dramatic impact on lowering the country’s maternal death rate (64). Before, almost 90% of births in Bangladesh occurred outside of hospitals; however, since the implementation of a mobile birth notification system dubbed “Mobile for Health,” approximately 89% of births have taken place within hospitals (65). There is a greater chance of preventing deadly diseases from spreading to mothers and children thanks to mobile phones and the ability to send text messages on vaccination initiatives. In addition, it is disseminated to notify the public about crucial precautions they can take to limit the spread of specific diseases. Information technology, as noted by Alataş (66), has a major effect on reducing the dangers of infant mortality by preventing pregnant women from skipping clinical appointments.

According to Zhao et al. (67) and Weili et al. (68), the ICT offer real-time feedback and are extremely useful for the decentralized health care system because of the development of novel health-related application. The advancement of healthcare has necessitated interdisciplinary understanding from fields as diverse as sociology, psychology, computer science, engineering, education, and others (69) in order to increase patient happiness with healthcare outcomes. Vaccination rates in many developing nations have increased as a result of this technology, which has been the subject of numerous research (70). Several professionals in the field feel that ICT has the potential to revolutionize the health care industry in the poor countries by making more relevant information more widely available. For instance, Liu et al. (71) described the state of the health care system in Uganda, where it was found that the providers of health services had saved about 24% of the total cost by employing information technology for the purpose of data collecting and storage (72–75). A number of studies have demonstrated that information and communication technology also serves as a tool to achieve the process of self-management. This is accomplished by easing the path of communication for elderly people, making it easier for these individuals to get in touch with their children, relatives, and medical professionals. According to Zhu et al. (76), the advent of ICT has provided people with greater chances to contact with others in healthcare services for the provision of illness management, clinical decision making, and other educational objectives. This enables individuals to work together to find solutions to their shared health issues.

## 3. Data and methodology

### 3.1. Data discussion

This study focuses on 25 provinces in China to investigate the core determinants of mortality rate. Therefore, this study uses the five determinants: carbon emissions, income inequality, health expenditures, green energy, and information and communication technology. Moreover, the selected variables data is collected from the Chinese yearbook. However, the health problems being measured by the mortality rate, infant per 1,000 live birth, carbon emissions in Kt, emissions (kt), economic inequality (Gini index), health expenditures measured by per capita medical care, green energy, and information and communication technology (ICT) by internet users.

**Figure 1:**
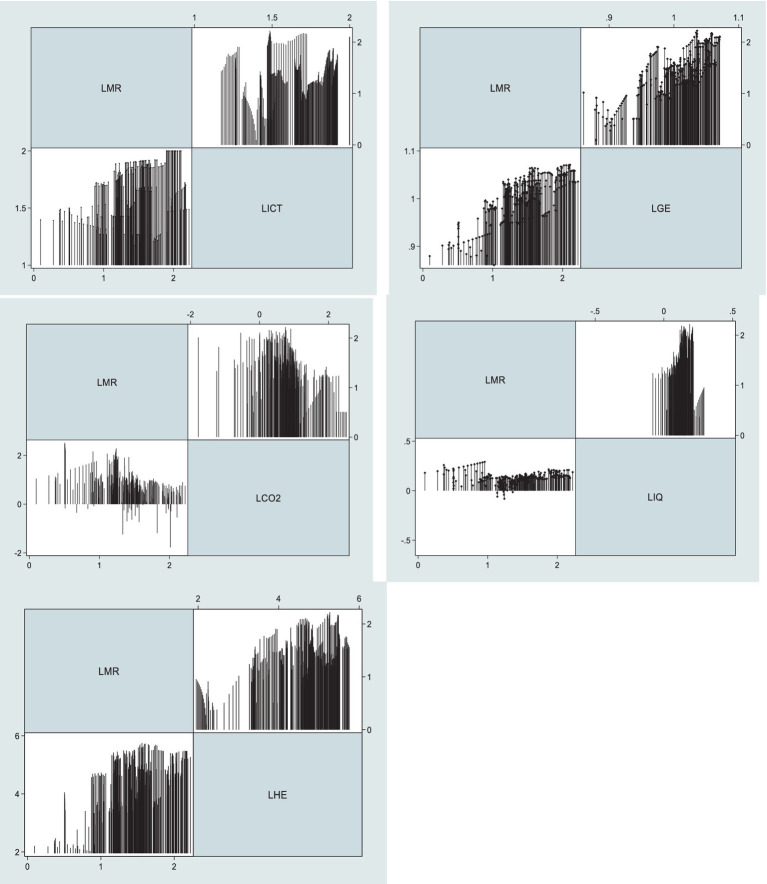
Graphical presentation of studied variables.

However, there may be a question of why authors selected the panel data rather than time series and cross-provinces. To answer this question, this study also tries to explain the fundamental logic behind the panel data selection. While cross-sectional and time-series data have their uses, panel data have a number of advantages due to their combination of inter-and intra-individual variances and dynamics. The effectiveness of econometric estimates is enhanced because panel data typically contain more degrees of freedom and higher sample variability than cross-sectional data, which may be seen as a panel with *T* = 1, or time series data, which is a panel with *N* = 1. Second, compared to single cross-section or time series data, panel data is more able to capture the nuances of human behavior. Thirdly, this incorporates at least two dimensions: a CS dimension and a time series dimension, simplifying computing and statistical inference. Compared to inferring from cross-sectional or time-series data, computing an estimator or deduction for panel data is expected to be more involved under typical conditions. However, there are situations where access to panel data streamlines calculations and draws more accurate conclusions. However, the data description is given in Table 1. Moreover, Figure 1 tries to explain the scatter graphs in a single diagram.

**Table 1:** Data description.

Variables	Unit	Source
MR	Mortality rate (infant per 1,000 live birth)	CYB
CO_2_	Carbon emission (kt)	CYB
IQ	Income inequality (Gini index)	CYB
HE	Health expenditure (per capita medical care)	CYB
GE	Green energy (% of total final energy)	CYB
ICT	Information and communication technology (internet users)	CYB

### 3.2. Model specification

One who is reading this could wonder why the economic disparity and the environmental impact are being employed in the same model. The public’s concern for environmental issues has been growing in recent years in response to developments such as the energy crisis, climate change, and the severity of other environmental problems. In developing countries, particularly China, rapid economic growth has resulted in excessive utilization of natural resources and has exacerbated the worsening of the ecological environment. This is especially true in China. The term “green consumption” refers to a behavior that is environmentally responsible and is characterized by promoting nature and preserving the ecology. In recent years, green consumption has attracted the attention of both businesses and consumers. The purchase of environmentally friendly goods for day-to-day use is widely recognized as a productive approach to addressing environmental concerns and can help reduce the risk of adverse health effects.

The income disparity in emerging economies is another reason why China’s government boosted health spending from $11 to $29 between 2000 and 2011. To provide universal health coverage, 80% of China’s health budget goes to public hospitals and clinics. While monetary gains are welcome, it is just as critical that individuals in need have access to health information and are literate in its use. We have argued that primary health education is an important factor in the positive relationship between healthcare practitioners and patients, from diagnosis to treatment. If everyone in a community has quick and simple access to health information, then everyone will be better equipped to make informed decisions about their health and take steps to reduce the prevalence of harmful behaviors and increase the prevalence of those that are beneficial to their health. Doctors and nurses need to improve their ability to communicate with all community members, not just patients. More and more health-related initiatives are taking place online and in communities, which further flattens our informational universe.

The study’s findings highlight the significance of income distribution and the damage it does to the environment. For its most basic form, Equation (1) can be written as follows:


(1) $${\text{HI}}_{i,t}=f{\left(\beta 0,\;\;{\text{CO}}_{2}{\;}^{\beta 1},\;\;{\text{IQ}}^{\beta 2},\;{\text{HE}}^{\beta 3},\;\;{\text{GE}}^{\beta 4},\;\;{\text{ICT}}^{\beta 5}\right)}_{I,t}$$

The above function can be transformed into a log–log model by using the natural log; In Equation (2), LMR, LCO_2_, LIQ, LHE, LGE, and LICT refers to the natural log of carbon emission, income inequality, health issue, health expenditure, green energy, and information and communication technology. This analysis is grounded on the basic regression model:


(2) $$\begin{array}{l} {\text{LMR}}_{i,t}=\beta_{0}+\beta_{1}\;{\text{LCO}}_{2i,t}+\beta_{2}\;{\text{LIQ}}_{i,t}+\beta_{3}\;{\text{LHE}}_{i,t}\\ \;+\beta_{4}\;{\text{LGE}}_{i,t}+\beta_{5}\;{\text{LICT}}_{i,t}+\varepsilon_{i,t} \end{array}$$

In Equation (2) “I” is for cross-sections, and “t” is the time period, such as from 2005 to 2020. β’s are the coefficients, L is the natural logarithm and ε is the error term.

However, another objective of this analysis is to investigate the moderate role of income inequality on health expenditure, green energy, and information and communication technology and their impact on the mortality rate. Similarly, the moderate effect Equations (3)–(5) are inserted below:


(3) $$\begin{array}{l} {\text{LMR}}_{i,t}=\beta_{0}+\beta_{1}\;{\text{LCO}}_{2i,t}+\beta_{2}\;{\text{LIQ}}_{i,t}+\beta_{3}\;{\text{LHE}}_{i,t}\\ \;+\beta_{4}\;{\text{LGE}}_{i,t}+\beta_{5}\;{\text{LICT}}_{i,t}+\beta_{6}\;{\text{LIQ}}^{*}\text{ HE}+\varepsilon_{i,t} \end{array}$$

This shows the moderate effect of IQ * HE on mortality rate and other variables remain the same in Equation (3).


(4) $$\begin{array}{l} {\text{LMR}}_{i,t}=\beta_{0}\;\beta_{1}\;{\text{LCO}}_{2i,t}+\beta_{2}\;{\text{LIQ}}_{i,t}+\beta_{3}\;{\text{LHE}}_{i,t}\\ \;+\beta_{4}\;{\text{LGE}}_{i,t}+\beta_{5}\;{\text{LICT}}_{i,t}+\beta_{6}\;{\text{LIQ}}^{*}\text{ GE}+\varepsilon_{i,t} \end{array}$$

This displays the moderate effect of IQ * GE on mortality rate and variables other than it remain as it is in Equation (4).


(5) $$\begin{array}{l} {\text{LMR}}_{i,t}=\beta_{0}+\beta_{1}\;{\text{LCO}}_{2i,t}+\beta_{2}\;{\text{LIQ}}_{i,t}+\beta_{3}\;{\text{LHE}}_{i,t}\\ \;+\beta_{4}\;{\text{LGE}}_{i,t}+\beta_{5}\;{\text{LICT}}_{i,t}+\beta_{6}\;{\text{LIQ}}^{*}\text{ ICT}+\varepsilon_{i,t} \end{array}$$

This displays the moderate effect of IQ * ICT on mortality rate and other variables continue the same in Equation (5).

### 3.3. Estimation strategy

#### 3.3.1. Cross-sectional dependence test

The cross-sectional dependence (CD) of Phillips and Sul (77) is vital for panel data since it might cause discrepancies and errors in study. Economic, political, social, and other channels such as bilateral trade and sharing boards are examples of relationships in the real world. These kinds of global relations may participate to the development of CD. To solve this problem, we use the CD test developed by Pesaran (78) and the LM test developed by Breusch and Pagan (79). The subsequent equation is used in CD testing to check for the existence of CD in the data.


(6) $$\text{CD}=\sqrt{\frac{2T}{N\left(N-1\right)\left(\sum_{J=1+1}^{N}Pji\right)}}$$

The period is expressed by T, and cross-sections by N. Here, we show how this equation can be used in an LM test to analyze panel data for CD.


(7) Yit=αi+βixit+εit

where T is the time range and I is the number of cuts made. Both of these approaches to estimation use the null hypothesis that cross-sectional dependency (CD) does not exist in the panel data, whereas alternative hypotheses take into consideration the existence of CD.

### 3.4. Cross-sectional unit root test

In addition, cross-sectional variation is assumed not to alter the results, which is not the case. As a result, Pesaran (80) came up with the CADF and CIPS models, which incorporate both the CS independence of Im et al. (81) and the CS augmentation of the Dickey-Fuller models. Both of these analyses account for cross-sectional and panel heterogeneity. In order to calculate whether or not the variables are consistent, second-generation unit tests have been used.


(8) $$\Delta x_{i,t}=\alpha_{i,t}+\beta ix_{i,t-1}+\rho_{i}T+{\sum^{n}}_{j=1}\;\theta_{i,t}\;\Delta x_{i}{,}_{t-j}+\varepsilon_{i,t}$$

where xit denotes the variable of interest, i shows the CS, t for the time window, and r shows the model’s residuals. Although the alternative hypothesis does account for stationarity, the null hypothesis does not.

### 3.5. Co-integration test

To the same extent as the first-generation panel unit root methods, the first-generation panel co-integration estimators do not take CD into account. The second-generation panel co-integration estimate proposed by Westerlund (82) can be used to ascertain the co-integrating features of the parameters in the existence of CD. Standard errors for the significance tests (Gt, Ga, Pt, and Pa) are evaluated using a bootstrapped method, which eliminates the CSD. Estimating Gt and Ga, both measures of group averages, allows us to compare the likelihood of two different hypotheses about the relationship between the variables across at least one cross-section. By contrast, the rigorous alternative hypothesis of co-integration across the series across all cross-sections predicts the two panel-mean statistics Pt and Pa.


(9) $$P_{t}=\beta /\text{SE }\left(\beta \right)$$

(10) $$P_{\beta }=T\left(\beta \right)$$

(11) $$G_{t}=1/M\;\sum_{i=1}^{m}\beta i/\text{SE }\left(\beta i\right)$$

(12) $$\text{Ga}=1/M\;\sum_{i=1}^{m}T\beta i/\beta i\;\left(1\right)$$

Equation (9) of Pt and Equation (10) of Pβ denote panel statistics whereas Equation (12) of Ga and Equation (11) of Gt denote group mean statistics. Here, no co-integration null hypothesis is evaluated.

However, the current study uses the most advanced and reliable estimators to investigate the proposed objective, i.e., CS-ARDL, AMG, and CCE-MG. In the light of conceptual and methodological contribution, these estimators have significant importance in dealing with panel data issues. However, the existing literature has used these estimators for development or environmental economics, while this study is being used for these estimators in the field of health economics. This is the leading gap that fills by the present study to existing literature. Moreover, the concerned techniques have advantages like cross-section dependence (CSD), endogeneity, heterogeneous slope coefficients, and non-stationarity, all controlled by these panel techniques.

#### 3.5.1. Cross-sectional augmented ARDL

Specifically, we use the Cross-Sectional Augmented Autoregressive Distributive Lag (CS-ARDL) model of Chudik and Pesaran (83) to examine the correlation between our variables. This model is an extension of the standard panel ARDL-PMG. The CS-ARDL framework takes into account both short-run and long-run factors, error correction factors, and the cross-sectional means of the relevant variables throughout both time periods. There aren’t many benefits to using this strategy instead of others. In the first place, it’s capable of giving trustworthy estimates even when factors are added in a non-intuitive order, as I(0) if I(0); otherwise, I(0) (1). In addition, it provides reliable data on the frequency of both acute and chronic stress disorders (83). Thirdly, the slope coefficients vary significantly from one another, making this an example of a mean group estimation. The CS-ARDL grounded on the mean group is an enhanced version of the ARDL model that uses CS averages as a proxy for the unnoticed common components and their lags rather than individual cross-sectional estimates (84). Last but not least, this method is useful when a weak exogeneity exists in the model as a result of a lagged dependent variable. In addition, the authors argued that the inclusion of the lagged cross-sectional averages in the model would mitigate the endogeneity issue. Following is the regression’s starting point model:


(13) $$\varphi CS-{\text{ARDL}}_{i}=\frac{\sum_{I=0}^{Pz}\beta pwi}{1-\sum I=0}{ϒ}_{I,i}$$

#### 3.5.2. AMG and CCEMG estimators

To deal with correlations, especially those between cross-sections, this empirical methodology makes use of the Common Correlation Effect Mean Group (CCEMG) technique created by Pesaran et al. (85). Despite the difficulties posed by the presence of diverse factors, this method is effective. Here is the CCEMG formula:


(14) $$\begin{array}{l} {\text{LMR}}^{i,t}=\alpha_{1}\;{\text{LCO}}_{2}{\;}^{i,t}+\alpha_{2}\;{\text{LIQ}}^{i,t}+\alpha_{3}\;{\text{LHE}}^{i,t}+\alpha_{4}\;{\text{LGE}}^{i,t}\\ \;+\alpha_{6}\;{\text{LICT}}^{i,t}+{ϒ}^{i}+W^{i,t}+\varepsilon^{i,t} \end{array}$$

α indicates cross-sectional coefficients, ϒ^i^ indicates constant for every CS, ε denoted error term and W is indicated cross-sections averages shown in Equation (14):


(15) $$\begin{array}{l} W^{i,t}=\alpha_{1}\;{\text{CO}}_{2}{\;}^{i,t}+\alpha_{2}\;{\text{IQ}}^{i,t}+\alpha_{3}\;{\text{HE}}^{i,t}\\ \;+\;\;\alpha_{4}\;{\text{GE}}^{i,t}+\alpha_{5}\;{\text{ICT}}^{i,t}+\alpha_{6}\;{\text{MR}}^{i,t} \end{array}$$

Equation (15) gives us the mean, but we need to account for time stupidity and patterns as well as the spillover effect and transient global or regional shocks. Additionally, the AMG estimator approach suggested by Wendt et al. (65) incorporates a common dynamic method into the country regression, which accounts for cross-sectional dependency (86). This estimator is a two-stage process:


(16) $$\text{Step}-1:\Delta y_{i,t}=\alpha_{i}+\beta_{i}\Delta x_{i,t}+{ϒ}_{i}\;f_{t}+\sum_{t}^{T}di\Delta D_{t}+\varepsilon_{i,t}$$

(17) $$\text{Step}-2:\beta_{\text{AMG}}=\frac{1}{N}\;\sum_{i=1}^{N}\beta_{I}$$

where Δy_i,t_ indicates the dependent variable; Δx_i,t_ denote the independent variable; β_i_ indicates country-exclusive estimation coefficients; f_t_ denotes the heterogeneous section is the unnoticed common factor; d_t_ denotes the standard dynamic process and time dummies’ coefficient; denotes β_AMG_ “the mean group estimator” for AMG; α_i_ and ε_i,t_ explain the intercept and error term, correspondingly. Furthermore, the robustness is examined by a mean group (MG).

## 4. Results and discussion

### 4.1. Descriptive statistics

In order to guide the additional empirical examination, it is necessary first to list descriptive statistics for the variable in question. This study focuses on LMR, LCO_2_, LIQ, LHE, LGE, and ICT. According to the given value, income inequality has the maximum mean value, and green energy has the lowest mean value. A significant difference does not exist between the mean and median, which indicates that there is no presence of an outlier in Table 2.

**Table 2:** Descriptive statistics.

Variables	Mean	Median	Maximum	Minimum	Std.
LMR	26,765	26,287	31,767	13,677	0.036
LCO_2_	23,544	22,954	55,265	16,287	0.053
LIQ	35,552	35,265	63,667	3,877	0.015
LHE	9,635	9,534	15,365	2,877	0.005
LGE	8,654	8,287	12,776	3,276	0.001
LICT	12,654	11,756	16,466	2,367	0.017

### 4.2. Pairwise correlation matrix

The strength and direction of the correlation between the variables determine whether there is a beneficial or adverse association between them. The correlation coefficient typically varies from minus one to plus one. Strong correlations have values close to +1, whereas weak ones have values closer to −1. In Table 2, we can see that the correlation coefficients between the independent variable are quite small, suggesting that there is no multicollinearity bias in the model estimation (Table 3).

**Table 3:** Correlation matrix.

Variables						
LMR	1					
LCO_2_	0.732*	1				
LIQ	0.472**	0.386*	1			
LHE	−0.653**	−0.536**	−0.637*	1		
LGE	−0.476*	−0.375*	−0.628**	−0.725**	1	
LICT	−0.637*	−0.476**	−0.548*	−0.217*	−0.703*	1

* and ** show the level of significance at 1 and 5%, respectively.

### 4.3. Cross-sectional dependency test

In particular, it presupposed that the CS are independent of one another. However, there may be CS reliance in the panel data as a result of globalization among nations. Ineffective and biased estimators could result from ignoring CSD during the estimate process. For this reason, CSD analyses were directed for this examination, and the outcomes are revealed in Table 4. Subsequently, CSD was identified as being present. Results from the first-generation unit root test (87) may be skewed and deceptive in the occurrence of CSD. This detection makes us handle the issue of CSD by employing a distinctive set of unit root tests, namely the second-generation scheme generated by Pesaran et al. (88), known as the CADF and CIPS.

**Table 4:** CSD test.

Variables	Pesaran’s test	Frees’ test	Friedman’s test
LMR	34,276 (0.012)	9,376 (0.000)	49,387 (0.001)
LCO_2_	42,466 (0.000)	31,377 (0.012)	48,987 (0.005)
LIQ	21,636 (0.000)	13,498 (0.031)	59,648 (0.023)
LHE	18,255 (0.001)	13,784 (0.029)	63,675 (0.013)
LGE	13,264 (0.021)	7,487 (0.000)	76,498 (0.005)
LICT	27,475 (0.005)	14,387 (0.002)	64,987 (0.000)

### 4.4. Results of CADF and CIPS unit root tests

IPS and LLC unit root tests, which are of the first generation, are not used in this empirical work since they do not account for the problem of CS dependence. Instead, we employ the CADF and the CIPS unit root tests. Crucially, the CIPS and CADF tests generate credible outcomes even when CS dependence and variation among sample nations are present. The results of the CADF and CIPS unit root tests reveal that the variables LCO_2_, LIQ, LHE, and LGE are integrated at the first difference, expected LMR and LICT. This research uses cutting-edge methods to achieve the desired results (Table 5).

**Table 5:** CADF and CIPS test.

Variables	CADF unit root test		CIPS unit root test	
	Level	1st difference	Level	1st difference
LMR	−5.588*	−7.474	−2.788*	−6.777
LCO_2_	−2.835	−5.835*	−1.565	−5.897*
LIQ	−1.286	−3.765*	−1.876	−5.756*
LHE	−1.676	−7.574*	−1.366	−3.876*
LGE	−1.873	−6.565*	−1.558	−3.497*
LICT	−2.764*	−6.566	−3.877*	−6.655

*, **, and *** means level of significance at 1%, 5% and 10%.

### 4.5. Panel co-integration approach

The ECM co-integration method established by Westerlund and Edgerton (82), was also used to successfully estimate the co-integration link between the two ED models. The outcomes of the co-integration tests are displayed in Table 6, which combines the constant and trend values. The H0 of no co-integration is rejected with bootstrapped significant *p*-values via panel and group statistics. This output demonstrates that the LMR, LCO_2_, LIQ, LHE, LGE, and LICT are all co-integrated. It follows that the null hypothesis (H0) of no co-integration between the relevant variables is rejected. Co-integration testing is obeyed by an analysis of the relationship between the variables. Consequently, co-integration necessitates the use of long-term estimators such as CS-ARDL and CC-EMG.

**Table 6:** Westerlund cointegration.

Statistics	Value	*Z*-value	*P*-value	Robust *P*-value
G_t_	−7.676	5.566	0.081	0.000
G_a_	−9.354	3.889	0.000	0.002
P_t_	−3.676	2.776	0.012	0.127
P_a_	−8.676	4.998	0.287	0.006

### 4.6. CS-ARDL estimator

Table 7 shows the outcomes of CS-ARDL that techniques only displays the short run and long run outcomes of the variables. However, the given table shows four different models, in first model this study shows the main effect by each variable on explained one. In models 2, 3, and 4 this study investigates the moderate role of income inequality on health expenditures (model 2), green energy (model 3), and LICT (model 4).

**Table 7:** Short and long run CS-ARDL estimator.

Variables	Model 1	Model 2	Model 3	Model 4
Short run result				
LCO_2_	0.7368** (0.012)	4.6478* (0.002)	0.9654* (0.005)	0.4677* (0.000)
LIQ	6.5275** (0.021)	0.6254** (0.012)	2.7665* (0.000)	0.5865* (0.001)
LHE	−0.7228* (0.000)	−7.9876* (0.000)	−0.6764* (0.000)	−0.2878* (0.001)
LGE	−8.2765** (0.028)	−0.8276** (0.021)	−0.9876* (0.000)	−0.2796** (0.005)
LICT	−0.5975** (0.031)	−0.2886** (0.028)	−0.6376* (0.000)	−0.4725* (0.001)
LIQ * HE	–	−0.6576* (0.000)	–	–
LIQ * GE	–	–	−0.3876** (0.006)	–
LIQ * ICT	–	–	–	−0.8287** (0.021)
Long run test				
LCO_2_	0.3876** (0.029)	4.4863* (0.000)	3.7765* (0.002)	0.7398* (0.005)
LIQ	0.9766* (0.000)	0.4376* (0.000)	6.9277** (0.012)	0.3987* (0.003)
LHE	−5.0365** (0.023)	−2.9656** (0.005)	−2.3872** (0.021)	−0.9376** (0.045)
LGE	−0.7387** (0.012)	−0.4346** (0.047)	−3.6353** (0.032)	−0.8367* (0.000)
LICT	−0.9275** (0.015)	−0.3987* (0.000)	−3.4776* (0.007)	−0.9375** (0.035)
LIQ * HE	–	0.3762** (0.045)	–	–
LIQ * GE	–	–	0.6487* (0.000)	–
LIQ * ICT	–	–	–	0.7665** (0.021)

*, **, and *** means level of significance at 1%, 5% and 10%.

Similarly, the given Short run results shows carbon emissions and income inequality increases the mortality rate, while health expenditures, green energy and ICT cause to decline in mortality rate. Moreover, in short run this study shows income inequality is the leading problem in mortality rate via its inverse association. Therefore, the long-term effects of carbon emissions on human health are positively influenced by a coefficient of 0.3876, 4.4863, 3.7765, and 0.7398%. According to numerous scientific investigations, CO_2_ worsens environmental conditions by increasing levels of air pollution, elevating health concerns (89–91). Heart sickness, lung cancer, stroke, lower breathing toxicities, chronic bronchitis, and early mortality are all linked to breathing in high quantities of contaminated air from emissions. This has led to several research connecting CO_2_ emissions to health problems (25). According to these findings, countries with significant environmental difficulties are more likely to have serious public health problems, such as cardiovascular and respiratory illnesses (92, 93). It’s consistent with previous research that found a favorable correlation between poor environmental quality and cardiovascular disease outcomes (94). Chen et al. (95) estimates also corroborate our findings, demonstrating that carbon pollution raises health concerns. In other words, it’s safe to assume that air pollution harms human health by lowering environmental quality, which in turn reduces productivity on the job. This effect is confirmed by the regression analysis, which uncovers a positive and statistically significant connection between emissions and health problems. Moreover, this regression result supports our primary aim by suggesting that health problems are increasing with CO_2_ emissions.

The findings indicate that income inequality contributes to higher rates of mortality. The results show that the death rate rises by 6.5275 percentage points in the short run (for every 1% rise in income disparity) and by 0.9766 percentage points in the long run (for every 1% rise in income inequality). According to our findings, unequal wealth distribution has negative effects on public health. The steep rise in wealth disparity in China has coincided with a corresponding rise in the mortality rate in China. There was very little risk of dying due to poverty levels in 1981. However, if wealth disparity continues to grow over the next 30 years, it could shave off around 0.56 years from men’s life expectancy and about 0.39 years from women’s. We take a traditional approach and simulate that income disparity does not straightly harm individual health to help estimate the life loss from income dissemination. This might not be the case. The latest estimates of a Gini coefficient of 0.5 (96) or even higher (97) in China suggest that we may be grossly underestimating the life loss from income disparity in current years. Although the relationship between individual health and income inequality is positive, as proposed by Khan et al. (28). Loss of life due to income inequality may affect population dynamics outside life expectancy, as the correlation between the two is stronger (98).

The results demonstrate that healthcare spending reduces life expectancy by −0.7228, −7.9876, −0.6764, and −0.2878% in the short term and −5.036, −2.965, −2.387, and −0.937%, respectively in the long run. Pavlichenko et al. (99) and Correia et al. (100) analyzed the research conducted in high-income nations and found that health insurance generally led to better health. To reestablish CMS, providing financial aid to the poor, and targeting the supply side, including strengthening the infrastructure of township health clinics, Newson et al. (101) studied the results of the World Bank Health VIII project in the province of Gansu in the northwest of China. When participants were asked to rate their health before and after the project, they discovered that it did not affect how they felt about their health. The results of an evaluation of a community-based health insurance plan implemented between 2003 and 2006 in one of China’s western provinces, done by Pavlichenko et al. (99), were good for the health status of enrollees. In contrast to the plan proposed by Pavlichenko et al. (99), NCMS initially did not pay for outpatient services in several areas. Until they are ill and qualify for inpatient service, patients may put off using health care services, which does nothing to lower the financial load on the enrolled or improve their health. He et al. (102) analysis using the same data as Pavlichenko et al. (99) supported the above result, suggesting that the provider payment mechanisms are distinct, with salary plus performance-based bonus versus fee-for-service (NCMS). High co-payments are another obstacle to healthcare access for low-income NCMS members, as He et al. (102) discovered.

Findings show that green energy is not without its detrimental effects on health problems. The outcomes illustrate that in the short term, increasing the use of green energy reduces health problems by −8.2765, −0.8276, −0.9876, and −0.2796%, and in the long term, by −0.7387, −0.4346, −3.6353, and −0.8367%. Here we feel the difference in coefficient because combinations of variables in the single model use. Simply put, this difference is due to the model differentiation via main and moderate role of income inequality. However, in the point of policymakers higher authorities must focus on their association rather than its impact. Therefore, it is surprising for us if higher authorities insist to use green energy it would bring a significant decline in mortality rate in long run. Pollution from carbon emissions is a major contributor to declining global health. China can lower its overall carbon emissions by favoring renewable energy sources in its manufacturing processes. As illustrated, REC is negatively influencing issues. Based on these results, countries adopting widespread usage of REC might expect significant improvements in health outcomes. To lessen the impact on people and the planet and to confirm a high standard of living and a healthy ecosystem, it is currently suggested that renewable energy sources be utilized to their fullest extent. If China makes better use of its RE sources, this could lead to a dramatic decline in health problems.

A similar, detrimental effect of LICT on mortality has been observed. The results reveal that in the short, and long term, the mortality rate decreases by 0.5975, 0.2886, 0.6376, and 0.4725% for every 1% increase in the level of information and communication technology, respectively. The most important takeaways from this study are as follows: First, and most importantly, our model confirms that there is a negative association between the rate of death and depth of the Internet’s development, popularity, infrastructure, information resources, and applications. Second, whereas the middle and western parts of China do not benefit as much from Internet expansion, the eastern part of the country does. Finally, the validated results for the transmission mechanism demonstrate that expanding Internet access has the potential to further lower mortality rates by encouraging improvements in science and technology, education, medicine, urbanization, and opening up. The major goal of this investigation is to look into the secondary influence of income disparity on health care costs, renewable energy, and information and communication technologies. In both the short and long term, income disparity is inversely related to mortality [this result is in line with Shah et al. (103)].

### 4.7. Robust check for CC-EMG estimation

The results of a rigorous test of the CCEMG estimation are shown in Table 8. Results from this model indicate that carbon emissions increase mortality by 0.7465, 0.4776, 0.5476, and 0.3987% at the 1, 5, and 10% levels of significance, correspondingly. Similarly, income inequality reduces the death rate by 0.4733, 0.8476, 0.4875, and 0.8465% points. Health expenditure has a negative influence on mortality rate, with a − 0.5976, −4.9873, −0.5765, and −0.2878% reduction in mortality for every 1% increase in health expenditure, respectively. Green energy is correlated with a decrease in mortality of −0.4665, −0.4878, −0.5774, and −0.3987%. A total of −0.5487, −0.5987, −0.6376, and −0.4997% decreases in mortality were seen after adjusting for the effects of LICT. Positive effects on mortality of −0.8765, −0.7076, and −0.6447% are also observed for the moderate effect.

**Table 8:** CC-EMG test.

Variables	Model 1	Model 2	Model 3	Model 4
LCO_2_	0.7465* (0.000)	0.4776* (0.005)	0.5476** (0.012)	0.3987* (0.005)
LIQ	0.4733* (0.001)	0.8476* (0.000)	0.4875* (0.002)	0.8465* (0.005)
LHE	−0.5976* (0.005)	−4.9873** (0.024)	−0.5765** (0.016)	−0.2878* (0.001)
LGE	−0.4665** (0.076)	−0.4878* (0.000)	−0.5774* (0.005)	−0.3987** (0.029)
LICT	−0.5487* (0.000)	−0.5987** (0.061)	−0.6376** (0.045)	−0.4997* (0.001)
LIQ * HE	–	−0.8765** (0.037)	–	–
LIQ * GE	–	–	−0.7076** (0.027)	–
LIQ * ICT	–	–	–	−0.6447* (0.000)

*, **, and *** means level of significance at 1%, 5% and 10%.

## 5. Conclusion and suggestions

The main emphasis of this analysis is the examination of determinants of mortality rate in 25 provinces of China for the period 2005 to 2020. These determinants are carbon emission, income inequality, health expenditure, green energy, and ICT. The focus on the China region is explained by its leading role in the world economy. The study first finds reliance through nations and CIPS and CADF panel unit root tests. Based on the validation of the co-integration association, then the study implements system CS-ARDL and CCEMG estimators that yield short and long-run CS-ARDL co-integration parameters for estimations. Moreover, this study employed the CCEMG test to investigate the robust check among the selected variables. The key results can be summarized as carbon emission and income inequality have a positive influence on the mortality rate. Health expenditure, green energy, and ICT have a negative impact on the mortality rate.

In light of these findings, economists and politicians are encouraged to propose policy actions that will boost ecological quality and lessen the load of health problems. First, the federal government, in conjunction with the state and provincial governments, should move swiftly to regularly maintain and appropriately manage the woods. Public investments in the forestry industry, including forest management, replanting, afforestation, and cleaning operations that engage locals in caring for the forest and providing rewards and motivations for their facilities in forest management, can help reduce CO_2_ emissions. In addition, the government should pass laws prohibiting deforestation and, if required, provide limited licenses to deforestation with the stipulation that twice as many trees be planted as are felled. In addition, shifting away from non-renewable energy and toward renewables in the energy sector is the most efficient way to lower carbon output. To immediately reduce air pollution, China could implement transportation reforms similar to those in the Netherlands, where wind power has replaced coal and oil as the primary source of electricity for trains. In addition, the government should mandate that all enterprises install pollution treatment plants. Lastly, the government should improve the quality of life for its citizens by investing in infrastructures like green spaces and sewage treatment plants. The ramifications of this approach will aid in managing health problems at the population level.

Moreover, our findings imply that relocation of wealth from the wealthy to the poor will enhance health outcomes and reduce health inequalities while also decreasing income inequality. State-run medical insurance programs (including the New Rural and urban Co-operative Medical Scheme etc.) and old-age insurance programs have been developed since the early 2000s (i.e., New Rural Old-age Insurance). This was the intended result of these initiatives. The rate of increase in China’s income gap appears to have slowed since about 2010 (26). To determine whether or if this is the start of a new tendency that would eventually improve the health of China’s population, more study is required.

The Chinese government has lately unveiled plans to advance the alternative-fuel vehicle sector. According to the plan, by 2035, electric, plug-in hybrid, and fuel cell vehicles (ICEVs) would account for just 50% of new vehicle sales in the country, while the remaining 50% will be traditional hybrids, which still rely fully on gasoline but offer greater fuel efficiency. Starting in 2035, new pure ICEVs will no longer be sold. Beginning in 2021, EV adopters will receive fewer monetary enticements than in the past; however, a new supportive strategy will emphasis on HFCVs to enhance the supply chain and technologies of the industry. In order to support the widespread use of AEVs in China, local governments are being urged to promote harmony between environmental and industrial interests. If rapid decarbonization of power generation occurs at the same time, China’s current AEV program will lead to decreased air pollution and carbon emissions from the automotive sector.

As a first step, we need a higher standard of internet application development in the health and medical domains. The growth of the Internet and its ancillary indicators has greatly facilitated the growth of medical treatment, which has greatly lowered the mortality rate and enhanced the health of citizens. Thereby, advancing “internet + medical” and “Healthy China 2030” need a solid groundwork for increased internet development in the medical and healthcare sectors. To further informatize the medical and health fields, the government should adopt regulations to stimulate the growth of online hospitals, establish an “internet + medical” standard, and encourage the expansion of internet infrastructure. The shortage of medical resources can be mitigated if hospitals make use of the Internet throughout the entirety of the disease diagnosis, prevention, and rehabilitation process. This encompasses the use of 5G networks, AI, and sensor networks to provide remote medical care. The health of patients can be better understood and information can be shared more easily among hospitals when they use EHRs.

## Data availability statement

Publicly available datasets were analyzed in this study. The details can be found in the article/Supplementary material.

## Author contributions

ZS: conceptualization, data curation, methodology, and writing—original draft. LD: data curation, visualization, supervision, editing, writing—review and editing, and software. All authors contributed to the article and approved the submitted version.

## Conflict of interest

The authors declare that the research was conducted in the absence of any commercial or financial relationships that could be construed as a potential conflict of interest.

## Publisher’s note

All claims expressed in this article are solely those of the authors and do not necessarily represent those of their affiliated organizations, or those of the publisher, the editors and the reviewers. Any product that may be evaluated in this article, or claim that may be made by its manufacturer, is not guaranteed or endorsed by the publisher.

## References

[1] Luo J Zhai S Song G He X Song H Chen J. Assessing inequity in green space exposure toward a “15-Minute City” in Zhengzhou, China: using deep learning and urban big data. Int J Environ Res Public Health. (2022) 19:5798. doi: 10.3390/IJERPH19105798 35627336 PMC9141614

[2] Chen F Wang M Pu Z. The impact of technological innovation on air pollution: firm-level evidence from China. Technol Forecast Soc Change. (2022) 177:121521. doi: 10.1016/J.TECHFORE.2022.121521

[3] Ngo TQ. How do environmental regulations affect carbon emission and energy efficiency patterns? A provincial-level analysis of Chinese energy-intensive industries. Environ Sci Pollut Res. (2022) 29:3446–62. doi: 10.1007/s11356-021-15843-w, PMID: 34389945

[4] Lin MC Wu CF. Transportation, environmental degradation, and health dynamics in the United States and China: evidence from bootstrap ARDL with a Fourier function. Front Public Health. (2022) 10:1894. doi: 10.3389/fpubh.2022.907390 PMC9277069 35844846

[5] Siddiqua A Hahladakis JN Al-Attiya WAKA. An overview of the environmental pollution and health effects associated with waste landfilling and open dumping. Environ Sci Pollut Res. (2022) 29:58514–36. doi: 10.1007/S11356-022-21578-Z PMC9399006 35778661

[6] Chien F Hsu CC Zhang YQ Vu HM Nawaz MA. Unlocking the role of energy poverty and its impacts on financial growth of household: is there any economic concern. Environ Sci Pollut Res. (2022) 29:13431–44. doi: 10.1007/S11356-021-16649-6, PMID: 34595698

[7] Sage N. Health and education: moving towards healthy human development Policy Press (2022).

[8] Sun Y Razzaq A Sun H Irfan M. The asymmetric influence of renewable energy and green innovation on carbon neutrality in China: analysis from non-linear ARDL model. Renew Energy. (2022) 193:334–43. doi: 10.1016/j.renene.2022.04.159

[9] Yang M Zhu H Li X Gong W Pang X Lv D. Study on the construction of a health lifestyle for older people in the Longevous area in China. Sustain. (2022) 14:12219. doi: 10.3390/SU141912219

[10] Wu CF Chang T Wang CM Wu TP Lin MC Huang SC. Measuring the impact of health on economic growth using pooling data in regions of Asia: evidence from a quantile-on-quantile analysis. Front Public Health. (2021) 9:689610. doi: 10.3389/FPUBH.2021.689610, PMID: 34532306 PMC8438143

[11] Wu CF Li F Hsueh HP Wang CM Lin MC Chang T. A dynamic relationship between environmental degradation, healthcare expenditure and economic growth in wavelet analysis: empirical evidence from Taiwan. Int J Environ Res Public Heal. (2020) 17:1386. doi: 10.3390/IJERPH17041386 PMC7068420 32098090

[12] Chien F Zhang YQ Sadiq M Hsu CC. Financing for energy efficiency solutions to mitigate opportunity cost of coal consumption: an empirical analysis of Chinese industries. Environ Sci Pollut Res. (2022) 29:2448–65. doi: 10.1007/S11356-021-15701-9, PMID: 34374014

[13] Xia Q. Does green technology advancement and renewable electricity standard impact on carbon emissions in China: role of green finance. Environ Sci Pollut Res. (2022) 30:6492–505. doi: 10.1007/s11356-022-22517-8 35997880

[14] Zang X. Policy regime change and environmental bill submission in China: evidence from provincial panel data (1992–2016). Int Public Manag J. (2022) 25:1051–71. doi: 10.1080/10967494.2022.2040663

[15] Wu D Song W. Does green finance and ICT matter for sustainable development: role of government expenditure and renewable energy investment. Environ Sci Pollut Res. (2022) 30:36422–38. doi: 10.1007/S11356-022-24649-3, PMID: 36547834

[16] Lister HE Mostert K Botha T van der Linde S van Wyk E Rocher SA. South African healthcare professionals’ knowledge, attitudes, and practices regarding environmental sustainability in healthcare: a mixed-methods study. Int J Environ Res Public Health. (2022) 19:10121. doi: 10.3390/ijerph191610121, PMID: 36011760 PMC9408692

[17] Xiong Q Sun D. Influence analysis of green finance development impact on carbon emissions: an exploratory study based on fsQCA. Environ Sci Pollut Res. (2022) 30:61369–80. doi: 10.1007/s11356-021-18351-z PMC8783589 35066850

[18] Asikha M Alam M Al-amin AQ. Global economic crisis, energy use, CO_(2)_ emissions, and policy roadmap amid COVID-19. Sustain Prod Consum. (2021) 26:770–81. doi: 10.1016/j.spc.2020.12.029, PMID: 33786357 PMC7994925

[19] Raza T Shehzad M Abbas M Eash NS Jatav HS Sillanpaa M. Impact assessment of COVID-19 global pandemic on water, environment, and humans. Environ Adv. (2023) 11:100328. doi: 10.1016/J.ENVADV.2022.100328, PMID: 36532331 PMC9741497

[20] Mallik A Chakraborty P Bhushan S Nayak BB. Impact of COVID-19 lockdown on aquatic environment and fishing community: boon or bane? Mar Policy. (2022) 141:105088. doi: 10.1016/J.MARPOL.2022.105088, PMID: 35529170 PMC9068432

[21] Rehman A. Radulescu M. Ahmad F. Kamran Khan M. Iacob S. E. Cismas L. M. (2022). Investigating the asymmetrical influence of foreign direct investment, remittances, reserves, and information and communication technology on Pakistan’s economic development. Available at: http://www.tandfonline.com/action/authorSubmission?journalCode=rero20&page=instructions

[22] Proskuryakova L. The interaction of environmental systems and human development in a time of wild cards. A big data enhanced foresight study. J Environ Manag. (2022) 316:115169. doi: 10.1016/J.JENVMAN.2022.115169, PMID: 35569357

[23] Khan H Khan I BiBi R. The role of innovations and renewable energy consumption in reducing environmental degradation in OECD countries: an investigation for innovation Claudia curve. Environ Sci Pollut Res. (2022):29:43800–13. doi: 10.1007/s11356-022-18912-w 35119641

[24] Ambika S Basappa U Singh A Gonugade V Tholiya R. Impact of social lockdown due to COVID-19 on environmental and health risk indices in India. Environ Res. (2021) 196:110932. doi: 10.1016/j.envres.2021.110932, PMID: 33647298 PMC7908889

[25] Rogers A Vassilev I Pumar MJJ Todorova E Portillo MC Foss C. Meso level influences on long term condition self-management: stakeholder accounts of commonalities and differences across six European countries health policies, systems and management in high-income countries. BMC Public Health. (2015) 15:622. doi: 10.1186/s12889-015-1957-1, PMID: 26152139 PMC4495781

[26] Burkle FM. Declining public health protections within autocratic regimes: impact on global public health security, infectious disease outbreaks, epidemics, and pandemics. Prehosp Disaster Med. (2020) 35:237–46. doi: 10.1017/S1049023X20000424, PMID: 32238221 PMC7156578

[27] Apergis N Mustafa G Dastidar SG. An analysis of the impact of unconventional oil and gas activities on public health: new evidence across Oklahoma counties. Energy Econ. (2021) 97:105223. doi: 10.1016/j.eneco.2021.105223

[28] Khan SAR Zhang Y Kumar A Zavadskas E Streimikiene D. Measuring the impact of renewable energy, public health expenditure, logistics, and environmental performance on sustainable economic growth. Sustain Dev. (2020) 28:833–43. doi: 10.1002/sd.2034

[29] Guo LH Cheng S Liu J Wang Y Cai Y Hong XC. Does social perception data express the spatio-temporal pattern of perceived urban noise? A case study based on 3,137 noise complaints in Fuzhou, China. Appl Acoust. (2022) 201:109129. doi: 10.1016/J.APACOUST.2022.109129

[30] Lin HH Hsu IC Lin TY Tung LM Ling Y. After the epidemic, is the smart traffic management system a key factor in creating a green leisure and tourism environment in the move towards sustainable urban development? Sustainability. (2022) 14:3762. doi: 10.3390/SU14073762

[31] Xiong Z Liu Q Huang X. The influence of digital educational games on preschool Children’s creative thinking. Comput Educ. (2022) 189:104578. doi: 10.1016/J.COMPEDU.2022.104578

[32] Oum S. Energy poverty in the Lao PDR and its impacts on education and health. Energy Policy. (2019) 132:247–53. doi: 10.1016/j.enpol.2019.05.030

[33] Qin LJ Chen CP Li YH Sun YM Chen H. The impact of the new rural cooperative medical scheme on the “health poverty alleviation” of rural households in China. J Integr Agric. (2021) 20:1068–79. doi: 10.1016/S2095-3119(20)63372-X

[34] Albrizio S Kozluk T Zipperer V. Environmental policies and productivity growth: evidence across industries and firms. J Environ Econ Manage. (2017) 81:209–26. doi: 10.1016/j.jeem.2016.06.002

[35] Mohsin M Abbas Q Zhang J Ikram M Iqbal N. Integrated effect of energy consumption, economic development, and population growth on CO_2_ based environmental degradation: a case of transport sector. Environ Sci Pollut Res. (2019) 26:32824–35. doi: 10.1007/s11356-019-06372-8, PMID: 31502046

[36] Murshed M. LPG consumption and environmental Kuznets curve hypothesis in South Asia: a time-series ARDL analysis with multiple structural breaks. Environ Sci Pollut Res. (2021) 28:8337–72. doi: 10.1007/s11356-020-10701-7, PMID: 33058083

[37] Anser MK Yousaf Z Nassani AA Abro MMQ Zaman K. International tourism, social distribution, and environmental Kuznets curve: evidence from a panel of G-7 countries. Environ Sci Pollut Res. (2020) 27:2707–20. doi: 10.1007/s11356-019-07196-2, PMID: 31836988

[38] Raza Z. Effects of regulation-driven green innovations on short sea shippinǵs environmental and economic performance. Transp Res Part D Transp Environ. (2020) 84:102340. doi: 10.1016/j.trd.2020.102340

[39] Caruso G Colantonio E Gattone SA. Relationships between renewable energy consumption, social factors, and health: a panel vector auto regression analysis of a cluster of 12 EU countries. Sustainability. (2020) 12:2915. doi: 10.3390/su12072915

[40] Titl V De Witte K. How politics influence public good provision. Socio Econ Plan Sci. (2021) 81:101000. doi: 10.1016/j.seps.2020.101000

[41] Kartal MT Samour A Adebayo TS Kılıç Depren S. Do nuclear energy and renewable energy surge environmental quality in the United States? New insights from novel bootstrap Fourier granger causality in quantiles approach. Prog Nucl Energy. (2023) 155:104509. doi: 10.1016/J.PNUCENE.2022.104509

[42] El Chaarani H Raimi L Brunei Darussalam U Seri Begawan B. Diversity, entrepreneurial innovation, and performance of healthcare sector in the COVID-19 pandemic period. J Public Aff. (2022) 22:e2808. doi: 10.1002/PA.2808

[43] Coccia M. Effects of the spread of COVID-19 on public health of polluted cities: results of the first wave for explaining the dejà vu in the second wave of COVID-19 pandemic and epidemics of future vital agents. Environ Sci Pollut Res. (2021) 28:19147–54. doi: 10.1007/s11356-020-11662-7, PMID: 33398753 PMC7781409

[44] Strelkovskii N Rovenskaya E Ilmola-sheppard L Bartmann R Rein-sapir Y Feitelson E. Implications of COVID-19 mitigation policies for national well-being: a systems perspective. Sustainability. (2021) 14:433. doi: 10.3390/SU14010433

[45] Roosa K Lee Y Luo R Kirpich A Rothenberg R Hyman JM. Short-term forecasts of the COVID-19 epidemic in Guangdong and Zhejiang, China: February 13–23, 2020. J Clin Med. (2020) 9:596. doi: 10.3390/jcm9020596, PMID: 32098289 PMC7073898

[46] Wu Y Jing W Liu J Ma Q Yuan J Wang Y. Effects of temperature and humidity on the daily new cases and new deaths of COVID-19 in 166 countries. Sci Total Environ. (2020) 729:139051. doi: 10.1016/j.scitotenv.2020.139051, PMID: 32361460 PMC7187824

[47] Bashir MF Ma B Bilal KB Bashir MA Tan D Bashir M. Correlation between climate indicators and COVID-19 pandemic in New York, USA. Sci Total Environ. (2020) 728:138835. doi: 10.1016/j.scitotenv.2020.138835, PMID: 32334162 PMC7195034

[48] Musah M Owusu-Akomeah M Kumah EA Mensah IA Nyeadi JD Murshed M. Green investments, financial development, and environmental quality in Ghana: evidence from the novel dynamic ARDL simulations approach. Environ Sci Pollut Res. (2022) 29:31972–2001. doi: 10.1007/s11356-021-17685-y, PMID: 35013976

[49] Khan SAR Ponce P Thomas G Yu Z Al-Ahmadi MS Tanveer M. Digital technologies, circular economy practices and environmental policies in the era of COVID-19. Sustainability. (2021) 13:12790. doi: 10.3390/su132212790

[50] Tariq S Mehmood U ul Haq Z Mariam A. Exploring the existence of environmental Phillips curve in south Asian countries. Environ Sci Pollut Res. (2022) 29:35396–407. doi: 10.1007/s11356-021-18099-6, PMID: 35048338

[51] Khan SAR Ponce P Yu Z. Technological innovation and environmental taxes toward a carbon-free economy: an empirical study in the context of COP-21. J Environ Manag. (2021) 298:113418. doi: 10.1016/j.jenvman.2021.113418, PMID: 34426217

[52] Chiu YB Lee CC. Financial development, income inequality, and country risk. J Int Money Financ. (2019) 93:1–18. doi: 10.1016/j.jimonfin.2019.01.001

[53] Hundie SK. Income inequality, economic growth and carbon dioxide emissions nexus: empirical evidence from Ethiopia. Environ Sci Pollut Res. (2021) 28:43579–98. doi: 10.1007/s11356-021-13341-7, PMID: 33840023

[54] Idrees M Majeed MT. Income inequality, financial development, and ecological footprint: fresh evidence from an asymmetric analysis. Environ Sci Pollut Res. (2022) 29:27924–38. doi: 10.1007/s11356-021-18288-3, PMID: 34982389

[55] Chen J Xian Q Zhou J Li D. Impact of income inequality on CO_2_ emissions in G20 countries. J Environ Manag. (2020) 271:110987. doi: 10.1016/j.jenvman.2020.110987, PMID: 32579533

[56] Galvin R. Yes, there is enough money to decarbonize the economies of high-income countries justly and sustainably. Energy Res Soc Sci. (2020) 70:101739. doi: 10.1016/j.erss.2020.101739, PMID: 32835010 PMC7424332

[57] Luo C Li S Sicular T. The long-term evolution of national income inequality and rural poverty in China. China Econ Rev. (2020) 62:101465. doi: 10.1016/j.chieco.2020.101465

[58] Huang R Tian L. CO_2_ emissions inequality through the lens of developing countries. Appl Energy. (2021) 281:116043. doi: 10.1016/j.apenergy.2020.116043, PMID: 33191970 PMC7651240

[59] Yang B Ali M Hashmi SH Jahanger A. Do income inequality and institutional quality affect CO_2_ emissions in developing economies? Environ Sci Pollut Res. (2022) 29:42720–41. doi: 10.1007/s11356-021-18278-5, PMID: 35088263

[60] Igawa M Managi S. Energy poverty and income inequality: an economic analysis of 37 countries. Appl Energy. (2022) 306:118076. doi: 10.1016/j.apenergy.2021.118076

[61] Baloch MA Danish Khan SUD Ulucak ZŞ Ahmad A. Analyzing the relationship between poverty, income inequality, and CO_2_ emission in sub-Saharan African countries. Sci Total Environ. (2020) 740:139867. doi: 10.1016/j.scitotenv.2020.139867, PMID: 32927531

[62] Liu Z Zhang H Zhang YJ Qin CX. How does income inequality affect energy efficiency? Empirical evidence from 33 belt and road initiative countries. J Clean Prod. (2020) 269:122421. doi: 10.1016/j.jclepro.2020.122421

[63] Saurabh S Dey K. Blockchain technology adoption, architecture, and sustainable agri-food supply chains. J Clean Prod. (2021) 284:124731. doi: 10.1016/j.jclepro.2020.124731

[64] Khan H Weili L Khan I. Examining the effect of information and communication technology, innovations, and renewable energy consumption on CO_2_ emission: evidence from BRICS countries. Environ Sci Pollut Res. (2022) 29:47696–712. doi: 10.1007/s11356-022-19283-y 35184242

[65] Wendt C Adam M Benlian A Kraus S. Let’s connect to keep the distance: how SMEs leverage information and communication technologies to address the COVID-19 crisis. Inf Syst Front. (2021) 1:1–19. doi: 10.1007/s10796-021-10210-z PMC8510881 34658661

[66] Alataş S. The role of information and communication technologies for environmental sustainability: evidence from a large panel data analysis. J Environ Manag. (2021) 293:112889. doi: 10.1016/j.jenvman.2021.112889, PMID: 34087642

[67] Zhao G Liu S Lopez C Lu H Elgueta S Chen H. Blockchain technology in agri-food value chain management: a synthesis of applications, challenges and future research directions. Comput Ind. (2019) 109:83–99. doi: 10.1016/j.compind.2019.04.002

[68] Weili L Khan H Khan I Han L. The impact of information and communication technology, financial development, and energy consumption on carbon dioxide emission: evidence from the belt and road countries. Environ Sci Pollut Res. (2022) 29:27703–18. doi: 10.1007/s11356-021-18448-5, PMID: 34984617

[69] Nirmal BC Singh RK. Contemporary issues in international law: environment, international trade, information technology and legal education. Singapore: Springer (2018).

[70] Magazzino C Mele M Morelli G Schneider N. The nexus between information technology and environmental pollution: application of a new machine learning algorithm to OECD countries. Util Policy. (2021) 72:101256. doi: 10.1016/J.JUP.2021.101256

[71] Liu K Ke F Huang X Yu R Lin F Wu Y. Deep BAN: a temporal convolution-based communication framework for dynamic WBANs. IEEE Trans Commun. (2021) 69:6675–90. doi: 10.1109/TCOMM.2021.3094581

[72] Ahamad S Gupta P Bikash Acharjee P Padma Kiran K Khan Z Faez Hasan M. The role of block chain techndology and internet of things (IoT) to protect financial transactions in crypto currency market. Mater. Today Proc. (2021) 56:2070–4. doi: 10.1016/j.matpr.2021.11.405

[73] Hou R. Chen Y. Wu J. Zhang H. (2015). A brief survey of optical wireless communication. Conferences in Research and Practice in Information Technology Series.

[74] Lin J Guo J Turel O Liu S. Purchasing organic food with social commerce: an integrated food-technology consumption values perspective. Int J Inf Manag. (2020) 51:102033. doi: 10.1016/j.ijinfomgt.2019.11.001

[75] Lu WC. The impacts of information and communication technology, energy consumption, financial development, and economic growth on carbon dioxide emissions in 12 Asian countries. Mitig Adapt Strateg Glob Chang. (2018) 23:1351–65. doi: 10.1007/s11027-018-9787-y

[76] Zhu Z Bai Y Dai W Liu D Hu Y. Quality of e-commerce agricultural products and the safety of the ecological environment of the origin based on 5G internet of things technology. Environ Technol Innov. (2021) 22:101462. doi: 10.1016/j.eti.2021.101462

[77] Phillips PCB Sul D. Dynamic panel estimation and homogeneity testing under cross section dependence. Econom J. (2003) 6:217–59. doi: 10.1111/1368-423X.00108

[78] Pesaran MH. Testing weak cross-sectional dependence in large panels. Econom Rev. (2015) 34:1089–117. doi: 10.1080/07474938.2014.956623

[79] Breusch TS Pagan AR. The lagrange multiplier test and its applications to model specification in econometrics. Rev Econ Stud. (1980) 47:239. doi: 10.2307/2297111

[80] Pesaran MH. A simple panel unit root test in the presence of cross-section dependence. J Appl Econ. (2007) 22:265–312. doi: 10.1002/jae.951

[81] Im KS Pesaran MH Shin Y. Testing for unit roots in heterogeneous panels. J Econom. (2003) 115:53–74. doi: 10.1016/S0304-4076(03)00092-7

[82] Westerlund J Edgerton DL. A panel bootstrap cointegration test. Econ Lett. (2007) 97:185–90. doi: 10.1016/j.econlet.2007.03.003

[83] Chudik A Pesaran MH. Common correlated effects estimation of heterogeneous dynamic panel data models with weakly exogenous regressors. J Econom. (2015) 188:393–420. doi: 10.1016/J.JECONOM.2015.03.007

[84] Chudik A Mohaddes K Pesaran MH Raissi M. Is there a debt-threshold effect on output growth? Rev Econ Stat. (2017) 99:135–150.

[85] Pesaran MH. Estimation and inference in large heterogeneous panels with a multifactor error structure. Econometrica (2006) 74:967–1012.

[86] Sadorsky P. Do urbanization and industrialization affect energy intensity in developing countries? Energy Econ. (2013) 37:52–59.

[87] Levin A Lin CF Chu CSJ. Unit root tests in panel data: asymptotic and finite-sample properties. J Econom. (2002) 108:1–24. doi: 10.1016/S0304-4076(01)00098-7

[88] Pesaran MH Ullah A Yamagata T. A bias-adjusted LM test of error cross-section independence. Econom J. (2008) 11:105–27. doi: 10.1111/j.1368-423X.2007.00227.x

[89] Facchini F Seghezza E. Public spending structure, minimal state and economic growth in France (1870–2010). Econ Model. (2018) 72:151–64. doi: 10.1016/j.econmod.2018.01.014

[90] Ramondt S Ramírez AS. Assessing the impact of the public nutrition information environment: adapting the cancer information overload scale to measure diet information overload. Patient Educ Couns. (2019) 102:37–42. doi: 10.1016/j.pec.2018.07.020, PMID: 30097378 PMC6289837

[91] Saghafipour A. Indirect and potential impacts of the covid-19 pandemic on the public health. J Res Health Sci. (2020) 20:e00492–2. doi: 10.34172/jrhs.2020.25, PMID: 33169724 PMC7585763

[92] Chen L Xu X. Effect evaluation of the long-term care insurance (LTCI) system on the health care of the elderly: a review. J Multidiscip Healthc. (2020) 13:863–75. doi: 10.2147/JMDH.S270454, PMID: 32922026 PMC7457853

[93] Rechel B. Funding for public health in Europe in decline? Health Policy. (2019) 123:21–6. doi: 10.1016/j.healthpol.2018.11.014 30509874

[94] Shi R Irfan M Liu G Yang X Su X. Analysis of the impact of livestock structure on carbon emissions of animal husbandry: a sustainable way to improving public health and green environment. Front Public Health. (2022) 10:835210. doi: 10.3389/fpubh.2022.835210, PMID: 35223746 PMC8873578

[95] Chen X Huang C Wang H Wang W Ni X Li Y. Negative emotion arousal and altruism promoting of online public stigmatization on COVID-19 pandemic. Front Psychol. (2021) 12:652140. doi: 10.3389/fpsyg.2021.652140, PMID: 34122237 PMC8187574

[96] Sarwar S Alsaggaf MI Tingqiu C. Nexus among economic growth, education, health, and environment: dynamic analysis of world-level data. Front Public Health. (2019) 7:1–15. doi: 10.3389/fpubh.2019.00307, PMID: 31709219 PMC6823185

[97] da Silva JMC de Castro Dias TCA de Cunha AC Cunha HFA. Public spending in federal protected areas in Brazil. Land Use Policy. (2019) 86:158–64. doi: 10.1016/j.landusepol.2019.04.035

[98] Office WHOEMR. COVID-19 public health emergency of international concern (PHEIC). Wkly Epidemiol Monit. (2020) 13:1.

[99] Pavlichenko A Smirnova D Susloparova D Syunyakov T Kostyuk G. A one-day cross-sectional study of antidepressants prescription patterns in public mental health services: clinical guidelines vs real clinical practice in Russia. Psychiatr Danub. (2021) 33:47–54. PMID: 34559778

[100] Correia S Luck S Verner E. Pandemics depress the economy, public health interventions do not: evidence from the 1918 flu. SSRN Electron J. (2020). doi: 10.2139/ssrn.3561560

[101] Newson L Bould K Aspin-Wood B Sinclair L Ikramullah Z Abayomi J. The lived experiences of women exploring a healthy lifestyle, gestational weight gain and physical activity throughout pregnancy. Health Expect. (2022) 25:1717–29. doi: 10.1111/hex.13514, PMID: 35514097 PMC9327828

[102] He L Mu L Jean JA Zhang L Wu H Zhou T. Contributions and challenges of public health social work practice during the initial 2020 COVID-19 outbreak in China. Br J Soc Work. (2022) 52:4606–21. doi: 10.1093/bjsw/bcac077

[103] Shah SAA Longsheng C. Evaluating renewable and sustainable energy impeding factors using an integrated fuzzy-grey decision approach. Sustain Energy Technol Assess. (2022) 51:101905.

